# Electrocardiographic abnormalities and associated factors among HIV-infected adults on antiretroviral therapy

**DOI:** 10.3389/frph.2024.1387464

**Published:** 2024-09-10

**Authors:** Zewudu Befkadu, Mohammed Ibrahim, Amanuel Tadelle, Elsah Tegene

**Affiliations:** ^1^ Department of Biomedical Science, College of Health Science, Mattu University, Mattu, Ethiopia; ^2^ Department of Biomedical Sciences, Institute of Health, Jimma University, Jimma, Ethiopia; ^3^ Department of Internal Medicine, Institute of Health, Jimma University, Jimma, Ethiopia

**Keywords:** cardiovascular diseases, ECG abnormalities, HIV-infection, Mettu, Ethiopia

## Abstract

**Background:**

Individuals living with HIV are at increased risk of developing cardiovascular diseases. This heightened vulnerability is influenced by various factors, including the direct impact of HIV infection, the side effects of HIV medications, and a higher presence of traditional cardiovascular risk factors. Detecting and managing cardiovascular diseases early in HIV-infected individuals is crucial for their overall health and well-being. Electrocardiography, a simple and non-invasive test, can provide valuable information in this regard. However, there is currently no published data on the prevalence of electrocardiographic abnormalities and the associated factors among HIV-infected adults in Ethiopia.

**Objectives:**

This study was aimed at assessing the prevalence of ECG abnormalities and associated factors among HIV-infected adults on antiretroviral therapy.

**Methodology:**

A hospital-based comparative cross-sectional study was conducted at Mettu Karl Specialized Hospital (MKSH), southwest Ethiopia, among 96 HIV-infected patients and 96 HIV-negative control groups. A systematic random sampling technique was used to select HIV-infected respondents, and HIV-negative respondents were purposively recruited from caregivers. A face-to-face interview with a semi-structured and pretested questionnaire was conducted to collect the socio-demographic and behavioral characteristics of the study participants. Electrocardiography was done for all study participants using a 12-lead electrocardiograph, interpreted by a cardiologist, and classified according to the Minnesota Code classification system. The data were entered into Epi-Data version 4.6 and exported to SPSS version 25 for analysis. Finally, descriptive statistics, chi-square, independent *t*-test, bivariable, and multivariable logistic regression analyses were done at a 5% significance level.

**Results:**

The study found that 49% of HIV-infected and 19.8% of HIV-negative participants had at least one ECG abnormality. The proportion of coded ST-segment abnormalities, T-wave abnormalities, longer QT interval, and sinus tachycardia was significantly higher in HIV-infected respondents than in HIV-negatives. Being a smoker [AOR = 3.7, 95%CI: 1.03–13.6], being on Protease inhibitors [AOR = 3.6, 95%CI: 1.02–13.1] and having CD4 less than 350 cells/mm^3^ [AOR = 3.2, 95%CI: 1.22–8.49] were significantly associated with ECG abnormalities among HIV-infected respondents.

**Conclusion:**

Compared to HIV-negative participants, HIV-infected patients had a significantly higher prevalence of ECG abnormalities. Screening for ECG abnormalities is needed for the early detection of cardiac abnormalities and the reduction of future complications.

## Background

Nowadays, individuals infected with HIV have a better chance of living longer with efficient antiretroviral therapy (ART) and treatment of opportunistic infections (OIs), opening the gate for non-AIDS-related comorbidities such as cardiovascular diseases (CVDs), which have emerged as prominent causes of morbidity and mortality in this population (1–3). Both direct effects of HIV infection and indirect effects from factors such as ongoing inflammatory response, aging, side effects of ART, or other patient factors can lead to cardiovascular complications (4). It is estimated that by the year 2030, 73% of HIV-infected individuals will be ≥50 years of age, and 78% of them will have CVD (5).

Studies also indicated that HIV-infected population has a two-fold higher risk of CVD and a 4.5-fold higher risk of sudden cardiac death (SCD) than HIV-negative people (6, 7). A study done in the United States showed that the proportion of mortality due to CVD in PLWH increased from 2% to 4.6% between 1999 and 2013 (8). The risk of CVDs such as acute myocardial infarction was reported to be up to 1.5 times higher in HIV-infected patients, with early occurrence at the average age of ten years younger than uninfected persons. This may be due to the accelerated progression of HIV-related atherosclerosis as a result of chronic systemic inflammation caused by viral infection (9–11).

While ART has revolutionized HIV treatment, it can also increase the risk of CVDs. This risk stems from ART-associated metabolic side effects like impaired glucose tolerance, unhealthy lipid profiles, insulin resistance, and body fat redistribution (12, 13). These metabolic changes can contribute to the development of premature atherosclerosis, a dangerous hardening of the arteries that paves the way for heart attacks and strokes (14). A study showed that HIV patients on ART had a two-fold increased risk of cardiovascular events than HIV-negative people (15). Furthermore, the risk was higher for protease inhibitors (PIs)-based therapy than for non-PIs containing treatments (16–18).

Cardiac diseases linked to HIV infection pose a serious public health threat due to their silent nature and high potential for death. Electrocardiography (ECG) provides a valuable tool for early detection and management of these hidden dangers in HIV-positive individuals, ultimately optimizing patient care and contributing crucial knowledge to the field. Several studies across diverse locations paint a clear picture that ECG abnormalities are prevalent among HIV patients. In Asian countries, reports indicate a range of 51%–54.5% prevalence (19–21), while studies in various African nations reveal a wider range of 34%–93% (22–26). Notably, the “Strategies for Management of Antiretroviral Therapy (SMART)” study demonstrated that any ECG abnormality served as an independent predictor of future cardiovascular events, highlighting ECG's potential as a convenient screening tool for HIV-positive individuals (27).

Ethiopia, a sub-Saharan African nation, carries a significant burden of HIV-related health challenges (28). While studies have explored cardiovascular risk factors in this population, no data exists on the prevalence of ECG abnormalities and associated factors among HIV-infected individuals in the country. To address this gap, our study aimed to assess the prevalence of ECG abnormalities and identify potential risk factors associated with them in HIV-positive adults receiving ART at Mettu Karl Specialized Hospital (MKSH) in southwest Ethiopia.

## Materials and methods

### Study design, period and area

A hospital-based comparative cross-sectional study was conducted at MKSH from January 11 to March 10, 2022. MKSH is located in Mettu Town, which is 600 km away from Addis Ababa, the capital city of Ethiopia, to the southwest. This hospital serves about 2.5 million people from different regions of the country. The hospital's health service covers the outpatient department, inpatient services, critical care, emergency intervention unit, and different clinics, such as antenatal care clinics, delivery services, tuberculosis clinics, ART clinics, and ophthalmology clinics. Currently, about 1533 HIV-infected adult clients are taking a different combination of ART drugs at this hospital.

### Population

#### Source population

##### Study group

All HIV-infected patients on ART at MKSH ART clinic.

##### Comparative group

Apparently healthy HIV-negative attendants who came to MKSH as caregivers.

#### Study population

##### Study group

All selected HIV-infected adults attending the ART clinic at MKSH during the study period and had no exclusion criteria.

##### Comparative group

All selected age and sex-matched, apparently healthy HIV-negative caregivers at MKSH during the study period.

### Eligibility criteria

#### Inclusion criteria

##### Study group

All HIV-infected patients aged ≥18 years and on follow-up in ART clinic, who were on ART for at least 6 months, and had a complete clinical profile during the data collection were included in the study group.

##### Comparative group

Age and sex-matched HIV-negative, apparently healthy caregivers in the hospital during the data collection period were included in the comparative group.

#### Exclusion criteria

##### Study group

Pregnant women and women in the postpartum period, patients taking drugs that have a direct effect on the cardiovascular system (except for ART drugs), severely ill patients who cannot respond or perform our study procedure, patients with history of known cardiovascular diseases, systemic hypertension, chronic respiratory diseases, diabetic mellitus, and chronic kidney disease were excluded from the study.

##### Comparative group

Pregnant women and women in the postpartum period, individuals taking drugs that have a direct effect on the cardiovascular system, individuals with positive HIV antibody test results during screening, individuals with history of known cardiovascular diseases, systemic hypertension, chronic respiratory diseases, diabetic mellitus and chronic kidney disease were excluded from the study.

### Sample size determination and sampling technique

#### Sample size determination

The sample size was determined by using STAT CALC Epi-info version 7.0.8.3 for two-population cross-sectional study, considering a two-sided confidence level of 95%, power of 80%, and a 1:1 ratio of HIV-infected and HIV-negative participants. Based on previous research in Ghana (24), we estimated the anticipated prevalence of ECG abnormalities to be 47% among HIV-positive individuals and 26% among HIV-negative individuals. With these parameters, the software determined a minimum sample size of 91 participants for each group. To account for potential non-response, we inflated the sample size by 10%, resulting in a total of 202 participants (101 HIV-infected and 101 HIV-negative).

#### Sampling technique

A systematic random sampling approach was employed to select participants living with HIV. Clinic data at the MKSH ART clinic revealed an average of 320 adult HIV-positive patients visiting per two months. Aiming for 101 participants, we calculated a sampling interval (K) of 3.17, rounding down to 3. This meant selecting every third patient visiting the clinic, starting with a randomly chosen first participant selected via lottery. If any selected patient didn't meet the inclusion criteria, they were replaced by the next eligible patient visiting the clinic. For the comparison group, we purposively recruited age- and sex-matched individuals who were HIV-negative and apparently healthy. These individuals were recruited from patient attendants at the hospital during the study period.

### Data collection procedures

Semi-structured and interviewer-administered questionnaires were used to collect data on socio-demographic and behavioral characteristics of the study participants. Data such as the duration of HIV disease since diagnosis, ART duration and regimen, viral load, CD4 count, and WHO clinical stage of HIV infection were obtained from the patient's medical record. Baseline clinical data were collected from medical records at the time of enrollment in ART programs.

### Anthropometric measurements

Participant height was meticulously measured using a stadiometer, without shoes, to the nearest centimeter. To ensure accuracy, each individual stood upright against the stadiometer with their head, shoulders, buttocks, and heels firmly in contact with the scale, maintaining a level gaze straight ahead. Weight was precisely recorded using a calibrated Seca scale (Seca gmbh & co. kg, Germany) positioned on a level surface. The scale's accuracy was verified daily with known weights. Each participant stood barefoot, wearing light clothing, and their weight was documented to the nearest 0.1 kg. Finally, the Body Mass Index (BMI) was calculated for each participant using the established formula: weight in kilograms divided by height in meters squared (kg/m^2^).

### Blood pressure measurement

Blood pressure (BP) was measured using a mercury sphygmomanometer and recorded to the nearest millimeter of mercury (mmHg). To ensure accurate readings, a standardized protocol was followed. Participants sat quietly for 5 min before the first measurement and again for 3 min before repeating it. The participant's arm rested comfortably with their palm facing up, supported at heart level, and legs uncrossed. They were encouraged to sit relaxed and comfortably. Two readings were taken from the right arm with a 3 min interval. If the difference between the two readings was less than 5 mmHg, the average was recorded as the final BP. For discrepancies exceeding 5 mmHg, a third measurement was taken, and the average of all three readings became the final BP.

### HIV testing for the comparative group

Trained healthcare workers conducted HIV testing on participants in the comparison group using a blood-based rapid diagnostic kit, following the national algorithm for HIV testing. This approach aligns with the World Health Organization's guidelines for provider-initiated HIV testing and counseling (PITC). Before testing, each participant provided verbal consent and received counseling adapted to the WHO's PITC guidelines (29). Only participants testing negative for HIV were included in the comparison group.

### Electrocardiography

A resting 12-lead body surface electrocardiography was performed using EDAN SE-1200Express electrocardiograph machine for each study participant. The ECG record was taken by a radiologist at a standardization of 1 mv = 10 mm and a paper speed of 25 mm/sec. Metallic ornaments and clothing above the waist were removed while respecting participant privacy. The skin was prepared for the electrode placement by shaving hair, rubbing with alcohol swabs, and the area dried with gauze pads. The cardiac gel was applied to electrode placement areas, and the electrodes were applied to the ankle of the lower legs, wrist of the forearms, and pericardial area. ECG was recorded in a quiet room with instructions to breathe normally, relax, remain calm and close eyes. The printed ECG record was reviewed and interpreted by a cardiologist and classified according to the Minnesota Code classification system (30).

### Study variables

#### Dependent variables

Electrocardiogram abnormalities such as arrhythmia, ST-segment abnormalities, T wave abnormalities, QT interval, conduction abnormalities, ventricular hypertrophy, QRS axis deviations, and pathologic Q wave were analyzed.

#### Independent variables

Sex, educational status, marital status, occupation, residence, alcohol use, smoking, chewing khat, physical activity, weight, height, and BMI, duration on ART, ART regimen, duration of HIV infection since diagnosis, CD4 count, viral load and WHO clinical stage.

### Operational definitions

• **ECG abnormality**- any abnormal ECG finding according to the Minnesota Code classification system.
• **Smoker**: Participants who had history of ever smoking or currently smoking.
• **Ever smoker:** Refers to individuals who had smoked for more than six months in their life time.
• **Current smoker**: Refers to the active smoking of one or more manufactured or hand-rolled tobacco cigarettes on one or more days within one month prior to the study.
• **Physical activity**: Any body movement produced by skeletal muscles causing energy expenditure (31).
• **Physically inactive:** Any vigorous physical activity <75 min or moderate physical activity <150 min per week (31).
• **Physically active: A**ny vigorous physical activities ≥75 min or moderate physical activities ≥150 min per week (31).
• **Recent CD4 count**: CD4 count conducted within the last 6 months prior to data collection.
• **Recent viral load:** viral load test performed within the last year prior to data collection.

### Data processing and analysis

Following data cleaning and coding, the information was entered into Epi Data (version 4.6.0.4) and exported to SPSS (version 25.0) for analysis. We explored the exported data to identify outliers and missing values. Categorical data were summarized as frequencies and percentages, with chi-square tests used to identify statistically significant differences between HIV-infected and HIV-negative groups. Continuous data were presented as means and standard deviations, with independent-samples *t*-tests employed to assess significant mean differences between the two groups. Bi-variable and multivariable logistic regression analyses were conducted to evaluate the influence of various factors on electrocardiographic findings. Variables exhibiting *p*-values < 0.25 in bi-variable analyses were included in the multivariable model. Backward selection was used to refine the multivariable model, and variables retaining *p*-values < 0.05 were deemed significantly associated with ECG abnormalities. The strength of association between independent and dependent variables was expressed using odds ratios with 95% confidence intervals.

### Data quality control

The initial English questionnaires were translated into Afan Oromo and Amharic by qualified professionals and then back-translated to ensure consistency with the original meaning. To optimize clarity and accuracy, a pre-test with 5% of the planned sample size was conducted at Bedelle Hospital. Feedback from this pre-test guided further questionnaire refinements.

Data collectors and supervisors received comprehensive training to ensure standardized data collection and enhance reproducibility. All equipment was calibrated before each measurement to guarantee precision. An independent cardiologist, blinded to participants’ HIV status, interpreted all electrocardiograms, minimizing potential bias.

## Results

### Socio-demographic characteristics of participants

A total of 202 study participants including 101 HIV-infected patients and age-sex-matched 101 HIV-negative individuals for comparison were interviewed and underwent electrocardiography. The ECG record of 10 study participants were excluded due to poor quality. The analysis included data from 96 participants in each group.

Sixty-seven (69.8%) of the study participants were females in both HIV-infected and HIV-negative group (*P* = 1). The mean age was 38.9 years (SD ± 9.4) for HIV-infected group and 38.3 years (SD ± 10.17) for HIV-negative individuals (*P* = 0.681), with a minimum age of 20 and a maximum of 64 years. A higher proportion of HIV-infected individuals lived in urban areas (77.1%) compared to the HIV-negative group (61.5%, *P* = 0.019). Forty-four (45.8%) of HIV-infected and 73 (76%) of HIV-negative participants were married (Table 1).

**Table 1:** Socio-demographic characteristics of study participants at MKSH, Mettu, southwest Ethiopia, 2022.

Socio-demographic characteristics		HIV-infected *N* = 96		HIV-negative *N* = 96		*P*-value
		*n*	%	*n*	%	
Sex	Male	29	30.2	29	30.2	1
	Female	67	69.8	67	69.8	
Age group	Less than 40	48	50	54	56.2	0.621
	40–59	42	43.7	38	39.6	
	60 and above	6	6.3	4	4.2	
Residence	Rural	22	22.9	37	38.5	0.019
	Urban	74	77.1	59	61.5	
Marital status	Single	11	11.5	16	16.7	<0.001
	Married	44	45.8	73	76	
	Divorced	28	29.2	4	4.2	
	Widowed	13	13.5	3	3.1	
Educational status	No formal education	14	14.6	16	16.7	0.213
	Primary	31	32.3	21	21.9	
	Secondary	30	31.3	27	28.1	
	Collage and above	21	21.8	32	33.3	
Occupation	Farmer	14	14.6	15	15.6	0.617
	Housewife	25	26	28	29.2	
	Merchant	11	11.5	12	12.5	
	Employed	25	26	29	30.2	
	Daily labourer	15	15.6	7	7.3	
	^a^Other	6	6.3	5	5.2	

SD, standard deviation.

^a^ Private, Students, female sex workers, no specified job.

### Behavioural characteristics and physical parameters of participants

Among the participants, 15.6% of HIV-infected and 10.4% of HIV-negative participants were smokers (*P* = 0.284). Similarly, there was no significant difference in the proportion of participants who reported alcohol drinking (13.5% in HIV-infected vs. 18.8% in HIV-negative; *P* = 0.327) or khat chewing (5.2% in HIV-infected vs. 10.4% in HIV-negative; *P* = 0.179) between the two groups. Physical activity levels were also comparable, with 53.1% of HIV-infected and 46.9% of HIV-negative participants categorized as physically active (*P* = 0.386).

The mean weight of HIV-infected and HIV-negative respondents were 60.6 ± 10.39 and 62.42 ± 6.5 kg respectively (*P* = 0.158), while their respective mean height was 1.66 ± 0.05 and 1.67 ± 0.06 meters (*P* = 0.087). Similarly, the mean BMI of HIV-infected and HIV-negative respondents were 21.99 ± 3.4 and 22.3 ± 2.04 Kg/m^2^ respectively (*P* = 0.435). The mean of diastolic blood pressure and systolic blood pressure in HIV-infected and HIV-negative participants were (73.59 ± 6.87 vs. 74.58 ± 7.52 mmHg; *P* = 0.343) and (112.92 ± 9.33 vs. 115.21 ± 9.51 mmHg; *P* = 0.094) respectively.

### HIV related characteristics of HIV-infected study participants

HIV-infected participants reported an average infection duration of 10.43 years (SD ± 4.22) and had been on ART for an average age of 8.87 years (SD ± 3.81). The mean recent CD4 cell count and viral load were 573.5 ± 278 cells/mm^3^ and 795.4 ± 2,216.4 viral copies/ml respectively, while the recent viral load was undetectable for 61% of HIV-infected participants. All HIV-infected participants were on ART medication, with 16.7% receiving regimens containing PIs (Table 2).

**Table 2:** BIO-clinical characteristics of HIV-infected participants at MKSH, Mettu, southwest Ethiopia, 2022.

Parameters		*n*	%
Recent viral load	<1,000 copies/ml	83	86.5
	≥1,000 copies/ml	13	13.5
Recent CD4 count	<350 cells/mm^3^	29	30.2
	≥350 cells/mm^3^	67	69.8
Duration of HIV infection since diagnosis	<5 years	12	12.5
	5–9 years	20	20.8
	≥10 years	64	66.7
Duration on ART	<5 years	15	15.6
	5–9 years	38	39.6
	≥10 years	43	44.8
ART regimen	PI containing	16	16.7
	Non-PI containing	80	83.3
WHO clinical stage	Stage 1	41	42.7
	Stage 2	24	25
	Stage 3	18	18.8
	Stage 4	13	13.5

ART, antiretroviral therapy; PI, protease inhibitors.

### Prevalence and types of ECG abnormalities

**Figure 1:**
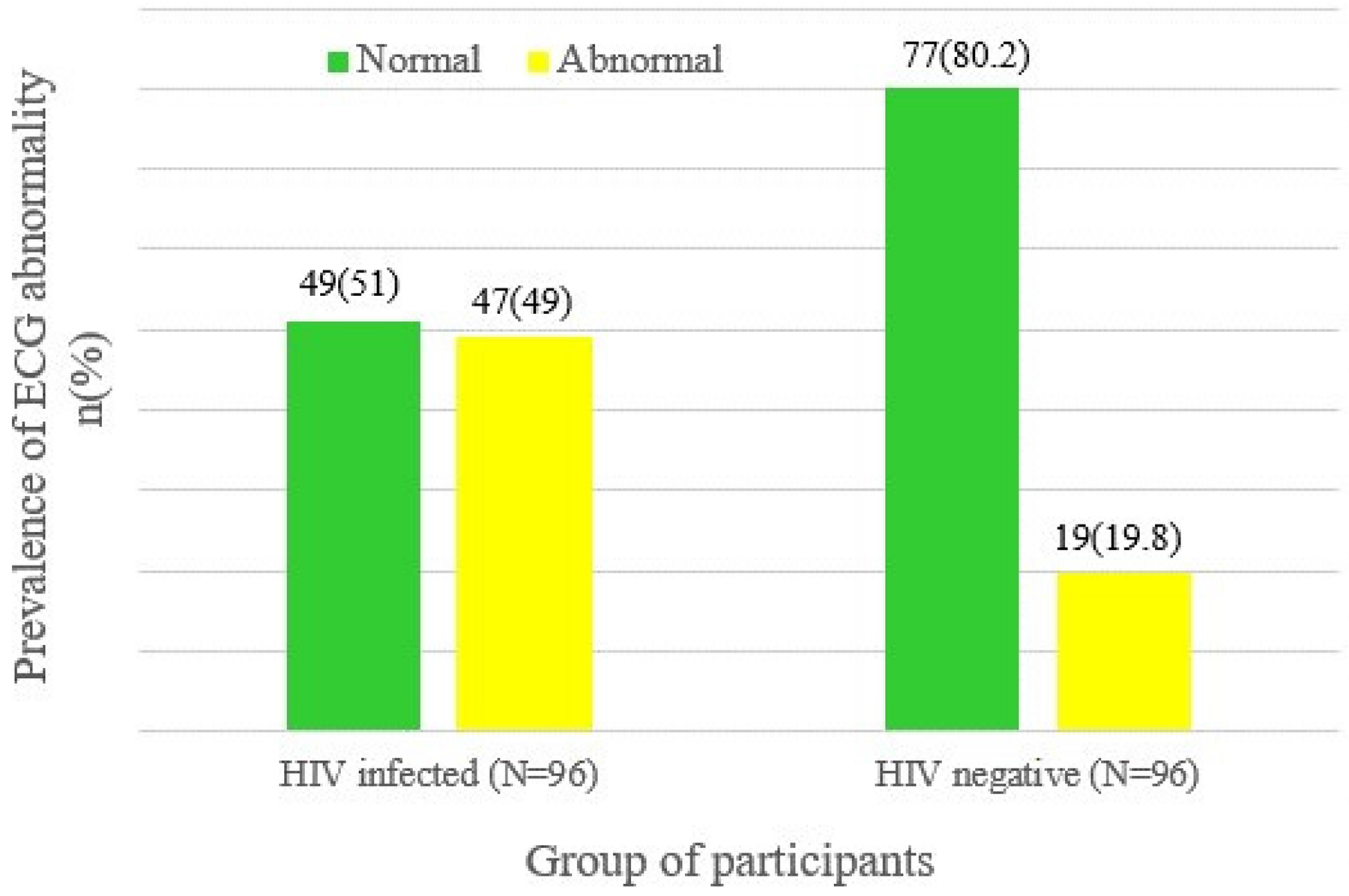
ECG status of HIV-infected and HIV-negative respondents at MKSH, southwest Ethiopia, Mettu, 2022.

In this study, 47 (49%) of the HIV-infected participants exhibited at least one ECG abnormality, compared to only 19 (19.8%) of the HIV-negative participants. This difference in prevalence was found to be statistically significant, indicating that ECG abnormalities are more common in HIV-infected individuals (*χ*^2^ = 18.1, *P* < 0.001**)** (Figure 1).

When comparing the types of ECG abnormalities, the prevalence of arrhythmia was similar between HIV-infected and HIV-negative participants. However, the mean heart rate was significantly higher in HIV-infected individuals. ST-segment abnormalities, T wave abnormalities, and increased QTc interval were significantly higher in the HIV-infected group compared to the HIV-negative group. Conduction abnormalities, ventricular hypertrophy and QRS axis deviations were not significantly different between the two groups. Other ECG abnormalities such as pathologic Q wave, low voltage QRS complex, poor R wave progression, and tall T wave were exclusively seen in HIV-infected individuals (Table 3).

**Table 3:** Types of ECG abnormalities among study participants at MKSH, mettu, southwest Ethiopia, 2022.

ECG abnormalities	HIV infected *N* = 96		HIV negative *N* = 96		*P*-value
Heart rate mean ± SD	80.77 ± 12.73		73.74 ± 9.29		^b^<0.001^a^
	*n*	%	*n*	%	
Any ECG abnormality	47	48.9	19	19.8	<0.001
Arrhythmia of any origin	12	12.5	7	7.3	0.227
Sinus tachycardia	9	9.4	2	2.1	0.030^a^
Sinus bradycardia	2	2.1	5	5.2	0.28
PVC	1	1	0	0	NA
Any conduction abnormality	7	7.3	2	2.1	0.169
1st degree AV block	3	3.1	2	2.1	NA
Right BBB	1	1	0	0	NA
Left BBB	1	1	0	0	NA
Left anterior FB	2	2.1	0	0	NA
Any axis deviation	1	1	2	2.1	NA
Left axis deviation	0	0	2	2.1	NA
Right axis deviation	1	1	0	0	NA
Ventricular hypertrophy	4	4.2	1	1	NA
RVH	2	2.1	0	0	NA
LVH	2	2.1	1	1	NA
ST-segment changes	13	13.5	4	4.2	0.022^a^
ST-depression	7	7.3	2	2.1	0.169
ST-elevation	6	6.3	2	2.1	0.279
Flat or negative T wave	10	10.4	3	3.1	0.044^a^
QTc interval in ms mean ± SD	428.99 ± 23.89	418.73 ± 22.54	^b^0.004^a^		
Other	4	4.2	0	0	NA

Other—pathologic Q wave, tall T wave, low voltage QRS, poor R wave progression.

^a^ Statistically significant.

^b^ Independent *t*-test; PVC, premature ventricular complexes; AV, aterio ventricular; BBB, bundle branch block; FB, fascicular block; VH, ventricular hypertrophy; QTc, heart rate corrected QT.

### Factors associated with ECG abnormalities among HIV-infected participants

Binary logistic regression was employed to determine factors associated with the ECG abnormality in HIV-infected participants. The final model fitness was checked by using Hosmer-Lemeshow goodness-of-fit test.

In bivariable logistic regression; residence, smoking status, physical activity status, HIV infection duration since diagnosis, ART regimen, recent viral load, recent CD4 level and WHO clinical stage of participants were associated with ECG abnormalities at a *P*-value of <0.25. Multivariable logistic regression using backward LR was done to identify the best predictors of ECG abnormalities. The collinearity of independent variables was checked by running pseudo linear regression, and fitness of the final model was evaluated by using Hosmer-Lemeshow goodness-of-fit test. Accordingly, the finding from multivariable logistic regression showed that smoking status, ART regimen and recent CD4 level were significantly associated with ECG abnormalities at a significance level of <0.05.

Compared to non-smokers, respondents who were smokers were 3.7 times more likely to have ECG abnormality (AOR = 3.7, 95%CI: 1.03–13.6). HIV infected respondents who were on PIs containing ART regimen had a 3.6 times higher chance of developing abnormal ECG findings than those who were on non-PIs containing ART regimen (AOR = 3.6, 95%CI: 1.02–13.1). HIV patients with recent CD4 cell count <350 cells/mm^3^ were 3.2 times more likely to have ECG abnormality than those with CD4 cell count ≥350 cells/mm^3^ (AOR = 3.2, 95%CI: 1.22–8.49) (Table 4).

**Table 4:** Bivariable and multivariable logistic analysis of factors associated with the ECG abnormalities among HIV-infected respondents at MKSH, Mettu, southwest Ethiopia, 2022.

Variable category	ECG abnormality		COR [95% CI]	*P*-value	AOR [95% CI]	*P* value
	Yes, *n* (%)	No, *n* (%)				
Residence						
Rural	8 (36.4)	14 (63.6)	1		1	
Urban	39 (52.7)	35 (47.3)	1.95 [0.73–5.2]	0.182	1.90 [0.64–5.68]	0.250
Smoking status						
Non-smoker	36 (44.4)	45 (55.6)	1		1	
Smoker	11 (73.3)	4 (26.7)	3.44 [1.10–11.71]	0.048^a^	**3.74 [1.03–13.6]**	**0**.**045**^a^
Physical activity status						
Active	22 (43.1)	29 (56.9)	1		1	
Inactive	25 (55.6)	20 (44.4)	1.65 [0.73–3.69]	0.226	0.84 [0.30–2.30]	0.735
HIV infection duration						
<5 years	3 (25)	9 (75)	1		1	
5–9 years	12 (60)	8 (40)	4.5 [0.92–21.92]	0.063	4.51 [0.84–24.1]	0.078
≥10 years	32 (50)	32 (50)	3.0 [0.74–12.11]	0.123	2.17 [0.48–9.66]	0.307
HAART regimen						
Non-PI containing	35 (43.7)	45 (56.3)	1		1	
PI containing	12 (75)	4 (25)	3.85 [1.14–12.99]	0.029^a^	**3.65 [1.02–13.1]**	**0**.**047**^a^
Viral load						
<1,000 copies/ml	38 (45.8)	45 (54.2)	1		1	
≥1,000 copies/ml	9 (69.2)	4 (30.8)	2.66 [0.76–9.34]	0.126	2.17 [0.52–9.06]	0.286
CD4 level						
≥350 cells/mm^3^	27 (40.3)	40 (59.7)	1		1	
<350 cells/mm^3^	20 (69)	9 (31)	3.29 [1.3–8.31]	0.012^a^	**3.21 [1.22–8.50]**	**0**.018*
WHO clinical stage						
Stage 1	14 (34.1)	27 (65.9)	1		1	
Stage 2	12 (50)	12 (50)	1.92 [0.69–5.93]	0.211^a^	1.76 [0.58–5.38]	0.316
Stage 3	12 (66.7)	6 (33.3)	3.85 [1.19–12.47]	0.024^a^	2.21 [0.37–13.2]	0.384
Stage 4	9 (69.2	4 (30.8)	4.33 [1.13–16.62]	0.032^a^	1.74 [0.17–17.2]	0.653

^a^ Statistically significant; COR, crude odds ratio; AOR, adjusted odds ratio; CI, confidence interval; HAART, highly active antiretroviral therapy; PI, protease inhibitors.

Bold value indicates variables that significantly associated with ECG abnormality.

### Association of HIV infection with ECG abnormality

After controlling for sex, smoking status, alcohol consumption, and physical activity, HIV infection was found to be independently linked to ECG abnormalities (AOR = 3.8; 95%CI: 1.99–7.38). HIV-infected individuals were 3.8 times more likely to experience ECG abnormalities compared to those without HIV infection (Table 5).

**Table 5:** Association of HIV infection with the ECG abnormalities, Mettu, southwest Ethiopia, 2022.

Variable category	ECG abnormality		COR [95% CI]	*P* value	AOR [95% CI]	*P* value
	Yes, *n* (%)	No, *n* (%)				
Sex						
Male	18 (31)	40 (69)	1		1	
Female	48 (35.8)	86 (64.2)	1.2 [0.64–2.39]	0.232	1.4 [0.65–3.1]	0.375
Smoking status						
Non-smoker	51 (30.5)	116 (69.5)	1		1	
Smoker	15 (60)	10 (40)	3.4 [1.43–8.10]	0.005^a^	3.3 [1.33–8.24]	0.010^a^
Alcohol drinking						
No	59 (36.6)	102 (63.4)	1			
Yes	7 (22.6)	24 (77.4)	0.5 [0.20–1.24]	0.136	0.5 [0.18–1.3]	0.143
Physical activity						
Active	37 (38.5)	59 (61.5)	1		1	
Inactive	29 (30.2)	67 (69.8)	1.4 [0.79–2.63]	0.225	1.6 [0.84–3.1]	0.147
HIV infection status						
Negative	19 (19.8)	77 (80.2)	1		1	
Infected	47 (49)	49 (51)	3.8 [2.04–7.38]	0.000	3.8 [1.9–7.38]	0.000^a^

^a^ Statistically significant; COR, crude odds ratio; AOR, adjusted odds ratio; CI, confidence interval.

## Discussion

This comparative cross-sectional study investigated whether ECG detectable cardiac abnormalities are more prevalent among HIV-infected study population compared to apparently healthy HIV-negative individuals, at MKSH, southwest Ethiopia. Overall, the present study found a significantly higher prevalence of ECG abnormality among HIV-infected patients than HIV-negative group. We also observed higher proportion of sinus tachycardia, ST-segment abnormalities, T wave abnormalities and longer QTc interval in HIV-infected group compared to HIV-negative one.

In the present study, 49% of HIV-infected participants had at least one type of ECG abnormality. This aligns with other studies conducted in Ghana (24), Nigeria (27), Zambia (22), northern India (32), and China (21). However, the prevalence observed here is lower than the studies done in Nigeria (26, 33) but exceeds studies done in Kenya (23), Cameroon (34), southern India (35), and China (36). The variations likely stem from differences in socio-demographic characteristics, distribution of cardiovascular risk factors, difference in clinical characteristics, and using different eligibility criteria.

This study found that HIV-infected individuals have significantly faster heart rates compared to HIV-negative controls. The mechanism underlying the sinus tachycardia could be related to the direct infection of autonomic ganglia, cardiomyocytes and neuroglia which is characterized by parasympathetic dysfunction in HIV patients (37). Elevation in proinflammatory cytokines in HIV infection was also linked with increased sympathetic nerve stimulations, and this might be attributed to increased heart rate in HIV patients (38, 39). In addition, the increased heart rate in the HIV-infected group might be due to anxiety, inter-current febrile illness, anaemia, myocarditis, and increased metabolic demand in HIV-infected patients (40). Our finding is similar to studies done in Nigeria (25) and Denmark (37) in which heart rate was reported to be higher among HIV-infected than HIV-negative respondents. Similarly, more prevalent sinus tachycardia in the HIV-infected group was reported in a comparative study done in China (36), cross-sectional studies done in India (19) and China (21).

Another notable finding in the present study was significantly higher ST-segment abnormalities in HIV-infected participants than in HIV-negative group. ST-abnormality could be caused by IHD, pericardial disease, or dilated cardiomyopathy that are known to occur frequently in HIV-infected individuals (41). HIV perpetuates the occurrence of IHD through inflammatory reaction in the coronary vessels which leads to endothelial dysfunction and early carotid artery atherosclerosis (38). Similar finding was also reported in studies done in Shanghai, China (36) and southeast Nigeria (33). On the other hand, our finding is in contrast to a comparative study done in Cameroon (34) which found ST-abnormality in only 1% of HIV-positive participants, and Nigeria (26) which reported no coded ST-segment abnormality in both HIV-infected and HIV-negative groups. The observed difference could be explained by differences in sociodemographic characteristics, sample size and inclusion criteria.

In the current study, T wave abnormalities including inverted or flat T waves were also another coded ECG abnormality which was significantly higher in HIV-infected respondents than HIV-negative comparative group. The present finding was comparable with comparative studies done in China and Nigeria (26, 33, 36). Flat or inverted T waves could be due to pericardial disease or dilated cardiomyopathy which are commonly seen in HIV-positive individuals (42).

The present study also found longer QTc interval in HIV-infected participants than HIV-negative controls. This finding is similar with study done in Thailand and USA (43, 44). Disturbance in cardiac electrophysiology which leads to delayed repolarization of cardiomyocytes could be the cause for this prolongation of QTc. It is proposed that HIV trans-activator protein called HIV Tat protein inhibits human ether-a-go-go-related gene coding for repolarizing potassium channels (hERGK^+^) which controls IKr K^+^ currents in cardiomyocytes (45). PIs could also block hERGK^+^ predisposing the patients to QT interval prolongation (18).

The present study showed that smoking is significantly associated with ECG abnormality among HIV-infected respondents. Toxic chemicals in cigarettes have profound effects on vascular endothelium, blood lipids, and clotting factors causing atherosclerosis which increase smokers’ risk for CVD (46). Nicotine, an active ingredient of tobacco, is also attributed to the release of catecholamines increasing the vulnerability to cardiac arrhythmias. It also contribute to CVD by affecting the myocardial oxygen demand-supply balance (47).

Being on PIs containing ART regimens was also one of the predictors of ECG abnormality in the current study. PIs are antiretroviral drugs that are known to have detrimental effects on the cardiovascular system. PIs may cause damage to cardiac myofiber physiology by increasing reactive oxygen species generation and resulting in endoplasmic reticulum stress. It also accelerates atherosclerosis by disturbing lipid metabolism (17). In addition, it was also suggested that PIs lead to blockage of repolarizing potassium current (IKr) channels, directly predisposing the subjects to QT interval prolongation (18).

The finding of the present study also revealed a decline in CD4 count below 350 cells/mm^3^ was associated with an increased incidence of ECG abnormalities. The relationship between the CD4 cell count and cardiac complications is not well understood. It might be mediated through the role of heightened inflammation and serum levels of inflammatory mediators, thereby predisposing them to endothelial dysfunction and atherosclerosis. In advanced HIV disease, depletion of the CD4 cell count leads to the activation of CD8 killer *T*-cells which mediate persistent immune dysfunction and inflammation (48).

Moreover, the current study revealed a significant association between HIV infection and ECG abnormalities even after adjusting for sex, smoking status, and physical activity. Individuals with HIV had a 3.8 times higher likelihood of displaying abnormal ECG results compared to those without HIV. This aligns with findings from previous studies in China (36) and Denmark (49), indicating that HIV infection is independently linked to ECG abnormalities, irrespective of traditional cardiovascular risk factors. The increased prevalence of ECG abnormalities among HIV-infected individuals may be attributed to factors such as persistent inflammation, atherosclerosis, dyslipidemia, and cardiac electrophysiological disturbances, which could stem from the direct impact of HIV, the side effects of ART, and other cardiovascular risk factors (14, 38, 45).

### Limitations of the study

Since it is cross sectional study, it is difficult to determine the precise relationship between variables and ECG abnormalities. In addition, biochemical tests such as serum electrolytes, lipid profiles and cardiac inflammatory markers were not done due to financial constraints. We were also unable to determine the actual time of HIV infection so the exact duration of HIV infection was not known.

## Conclusion and recommendation

The study found that ECG abnormalities were more prevalent in HIV-infected individuals compared to the HIV-negative group, with one out of every two HIV-infected participants exhibiting at least one abnormality. Independent risk factors for these abnormalities included smoking, being on antiretroviral therapy regimens containing protease inhibitors, and a decline in CD4 count. Based on these findings, it is recommended that healthcare providers screen all HIV-infected patients for ECG abnormalities, paying particular attention to those with a history of smoking, those taking protease inhibitors, and those with low CD4 counts. This will facilitate the timely identification of heart abnormalities and enable the administration of the required intervention prior to further cardiac complications. Additionally, future research should consider conducting longitudinal studies with larger sample sizes and incorporating biochemical tests to better understand the causal relationship between HIV infection and ECG abnormalities.

## Data Availability

The original contributions presented in the study are included in the article/Supplementary Material, further inquiries can be directed to the corresponding author.
